# The school of hard knocks: systemic violence and the motivation to harm in boys’ youth academy football

**DOI:** 10.3389/fsoc.2025.1631118

**Published:** 2025-08-25

**Authors:** Nick Gibbs, Daniel Briggs

**Affiliations:** Northumbria University, Newcastle upon Tyne, United Kingdom

**Keywords:** football (soccer), systemic violence, youth academy, motivation to harm, youth football (soccer)

## Abstract

The ‘beautiful game’ of football may seem to be a curious artifact of study for this scholarly collection on violence. However, this article will highlight the need to explore the boys’ English youth academy (YA) football industry as a manifestation of systemic violence and, ultimately, a reflection of the pseudo-pacified neoliberal economy. Embedding our theoretical analysis within emerging literature on harm and violence, this paper will illuminate the dark underbelly of boys’ elite-level youth football in England, examining the culture and relationships between academy players, YAs as breeding grounds for neoliberal subjectivities, the common practice of granting false hope to a ‘supporting cast’ of boys, and the underpinning inequalities in the elite academy industry. Drawing on data gleaned from thirty-five semi-structured interviews with current professional football practitioners and officials, as well as two former YA players, the work will provide a multifaceted analysis of the baked-in violence of the boys’ youth academy system. We will argue that we ought to challenge the assumption of harmlessness that currently cloaks the systemic violence of the boys’ elite game and move beyond *interventionitis*, in favor of wholesale change.

## Introduction

The ‘beautiful game’ of football captivates a global following of billions each year, inspiring children from across the world to adopt variations of ‘jumpers for goalposts’ to mimic their favorite sporting superstars. Yet the story of football’s commercial success is one of global domination and, whilst those of us alive to the sporting press are well-versed in critiques of sportswashing, match fixing, and morally bankrupt players, we often suppress knowledge of these ills in service of a view that the sport is a force for good (Black et al., 2024).

Why then has this article found a home in a special issue on violence? And how can the development of players in the sport that is the lifeblood of many be considered systemically violent? Indeed, whilst the presence of what Slavoj Žižek refers to as subjective violence is well-trodden turf in relation to football hooliganism (Hopkins and Treadwell, 2014; Atkinson, 2022), domestic abuse (Brooks-Hay and Lombard, 2018) and child sexual abuse (Dixon, 2020), it is not physical violence that we will study here. Instead, our gaze is trained on the elite boys’ youth academy (YA) system, where the next generation of male footballers are coached and educated. More specifically, we are interested in exactly how the brutalities of the notoriously cutthroat elite football industry manifest for the young lads who are trying to make it in the game. To this end, we will lean on Žižek’s (2008) tripartite theory of violence alongside Hall’s (2000) concept of pseudo-pacification to ask the questions: *what does elite boys’ football talent development look like under neoliberalism? And what forms of systemic violence are the children exposed to during their pursuit of the seductive dream of becoming a professional footballer?*

We therefore seek to move beyond the simplistic notion that the academy system’s necessary selectivity, in the form of ‘release’ for those who do not show the requisite mental or physical attributes, is its defining feature in relation to violence. Whilst this denial of desire is of course profound, existing work has already been undertaken by various scholars on this matter (Brown and Potrac, 2009; Blakelock et al., 2016; Blakelock et al., 2019; McGlinchey et al., 2022; Gorman and Blackwood, 2025). Instead, we wish to develop on our previous critique of the English boys’ YA industry (Gibbs and Briggs, 2025) to first explore the interrelationships between academy boys and the cultivation of neoliberal subjectivities, before analysing the common practice of granting false hope to a ‘supporting cast’ of players who are only retained to facilitate the growth of a handful of genuine footballing prospects. Cumulatively, this will facilitate a more holistic examination of the inequalities that underpin the elite academy industry.

What we set out to do therefore falls under the auspices of critical sports criminology (‌Groombridge, 2016; Silva and Kennedy, 2022a; Millward et al., 2022; DeKeseredy, 2025). As documented by DeKeseredy (2025), criminological accounts of the dark side of sport have, until recently, been conspicuously absent from the discipline. Though sport has long been considered as a site for criminality, especially in scholarship on football hooliganism, the advent of critical sports criminology allows us to exhume ‘the ways in which sport and sporting culture contributes to and reflect problematic, harmful, and potentially misleading discourses and understandings of crime, deviance, and the criminal justice system’ (Silva and Kennedy, 2022b: p. 11). More than this, as the sub-discipline has grown to encompass zemiological perspectives, social harms inherent in practices like the maltreatment of migrant workers in the FIFA World Cup (Millward et al., 2022) have highlighted the utility of critical sports criminology in unpacking the political economy of the elite football industry. Similarly, Gallacher’s (2019, 2022) investigations of the harms of youth football act as a blueprint for this article as we aim to build from the foundations she has laid in relation to systemic violence and the children’s game. Ultimately, this article responds to ‌Millward et al.’s (2022: p. 3) call for ‘bridge-building between sport and critical criminology’ by widening the criminological imagination (Young, 2011) and shedding light on a previously unexplored sporting setting.

### Introducing the English youth academy system

The Premier League (2011: p. 5) defines a youth academy as ‘the training environment operated by a professional football club for the development of youth players’, structured within a four-tier classification system known as ‘Categories’. Clubs are designated as Category One, Two, Three, or Four based on their level of resource investment (EFL, 2024), with Category One YAs being ascendant. Categories One to Three can recruit players as young as under-9 s, while Category Four clubs focus on developing players aged under-17 to under-21 (Premier League, 2011). Unsurprisingly, well-funded clubs like Manchester City and Chelsea FC occupy Category One, whereas smaller clubs are more prevalent in the lower categories.

Currently, over 90 English clubs operate licensed academies (Premier League, 2022). However, these institutions are highly selective by design (Champ et al., 2020), with Calvin (2017) estimating that only 180 out of the 1.5 million children playing organised football in England become a Premier League professional. Wilkinson (2021: p. 859) describes this process as ‘fundamentally destructive’, noting that 99% of children fail to progress, and 85% of those holding scholarships at age 16 do not earn professional contracts. To address these challenges, the academy system has undergone significant reform through the Elite Player Performance Plan (EPPP). Introduced in 2012 by the Premier League after a review of the national team’s shortcomings (O’Gorman et al., 2021), the EPPP focuses on four pillars: the Games Programme, Education, Coaching, and Elite Player Performance (Premier League, 2024). During its first decade, the new framework saw full-time academy coach numbers grow from 250 to 800, and a £1.94 billion investment in youth development by the English Premier League (EPL) and its clubs (Premier League, 2022).

Category One to Three academies now operate a three-phase model: Foundation (ages 5–11), Youth Development (ages 12–16), and Professional Development (ages 17–21) (Bullough and Jordan, 2017). Players at Category One academies may receive up to 8,500 h of coaching (Tears et al., 2018), and all Category One to Three academies provide post-16 education. However, the costs are substantial, with annual expenses for a Category One programme ranging from £2.3 million to £4.9 million (Larkin and Reeves, 2018), though these are funds that clubs can more than recoup in the longer term. For example, between 2014 and 2023, Chelsea FC earned £347 million from youth player sales, followed by Tottenham Hotspur at £256 million, and Manchester City at £254 million (CIES, 2024). Academies, therefore, are big business.

### Systemic violence and the motivation to harm

This paper represents the first attempt to understand the youth academy football industry through the prism of violence. In Žižek’s (2008) tripartite theory, he sets out three overarching types of violence: subjective, symbolic, and systemic. Žižek understands *subjective* violence as interpersonal physical confrontation, violence performed by ‘an identifiable agent against an identifiable victim’ (Raymen, 2023: p. 53). Picture, for example, the fracas between rival firms during the height of football hooliganism in the 1980s (Gibbons et al., 2008). *Symbolic* violence, on the other hand, is violence that takes the form of language and discourse. In footballing terms, the abuse of match officials could be categorized as such (Webb et al., 2020) or indeed the prevailing issues of racism and hate crime in the game (Awan and Zempi, 2023).

However, our central concern in this paper is Žižek’s interpretation of *systemic* violence. This term describes the embedded violence intrinsic to the political economic system which is necessary for the smooth functioning of late capitalism. Žižek inverts the common assumption that violence is fundamentally out of the ordinary and instead places systemic violence as a generative aspect of our normality (see Lynes et al., 2024). Put more simply, for neoliberalism to function, certain brutalities and inequalities must play out, causing harm to those who are subject to the system. Hall (2000, 2012a, 2012b) has consequently argued that, as society has developed since the Middle Ages, the level of violence has remained, but the character of that violence has broadly been converted from subjective to objective (played out as systemic and symbolic violence) (Horsley et al., 2015). This transmogrification, according to Hall and Winlow (2015: p. 116), was necessary to stimulate trade in the form of ‘functionally aggressive but physically pacified’ violence, manifest as competitive individualism, economic self-interest, and the demise of altruism. It is this form of violence that we are interested in here. More specifically, we will interrogate how the systemic violence that animates the political economy plays out in the YA setting.

Crucial to this analysis is an understanding of the positive and negative motivations to harm (Hall and Winlow, 2015). The positive motivation to harm describes the subjective inclination to inflict harm on others (Lloyd, 2018; Kotzé, 2024). On the other hand, the negative motivation to harm can be understood as when ‘unintentional harm to communities and individuals is engendered by the *normal functioning of capitalism*’ (Telford and Lloyd, 2020: p. 596, italics in original). This motivation is therefore a byproduct of the political economy’s smooth running and the repercussions of decisions made by those committed to it. These motivations do of course overlap but this crude dichotomy allows us to interrogate the motivation as well as effect of systemic violence on those in the YA system.

Building on our recent paper which outlined several socially harmful elements of the YA industry (Gibbs and Briggs, 2025), we aim to move beyond existing critiques of elite boys’ football and instead pose the following questions:

- W*hat does elite boys’ football talent development look like under neoliberalism?*
- *What forms of systemic violence are the children exposed to during their pursuit of the seductive dream of becoming a professional footballer?*

## Methodology

This work is built upon thirty-five semi-structured interviews conducted with practitioners in the boys’ youth academy industry, alongside two semi-structured interviews with former YA players (one of whom is now a professional footballer, and the other was released from a Category Three YA in 2016). Participants were purposively sampled via personal networks and the professional networking platform LinkedIn (see Dicce and Ewers, 2021), where keyword searches identified individuals currently employed in the boys’ English YA sector. From this, experts in areas such as academy player care, coaching, leadership, education, consultancy, governance, and recruitment were contacted, resulting in interviews with professionals from Category One, Two, and Three clubs, a national governing body, several charities, and a player care consultancy firm. Each participant signed a consent form and pseudonyms have been used to ensure participant and institutional confidentiality. As has been noted elsewhere (‌Moore and Stokes, 2012; Law, 2019), gaining research access into the elite football industry is often hampered by participants’ political and reputational concerns. However, given that this work was internally funded, we were able to approach participants as independent researchers, not affiliated to any club or governing body. The use of LinkedIn bolstered this as, rather than contact clubs or organizations formally, we were able to reach relevant individuals ‘through the side door’ using their online professional profiles.

Reflecting the existing research on the lack of gender and racial diversity in elite youth football coaching (Bradbury and Conricode, 2020, 2024), most of the sample were male (*n* = 30) and identified as white (*n* = 29). Levels of YA experience ranged from 6 months to 22 years, with representation from entry grade to senior leadership. Interviews, lasting (rather fittingly) between 45 and 90 min, were undertaken in-person and online before being transcribed and analysed thematically. Ethical approval was granted by Northumbria University’s Ethics Panel.

Finally, it is worth noting researcher positionality. Though, unlike some YA scholars (Champ et al., 2020; McGlinchey et al., 2022), neither of us are ‘insiders’, we both speak fluent football and began this project from an informed fan and amateur player perspective. Moreover, the first author’s support of a team boasting a Category Two academy in the English fourth division underpins a passionate interest in youth development. This shared obsession for the beautiful game frames the following analysis as, far from being football detractors, we wish to see the youth system and its players flourish beyond the constraints and challenges that we will describe.

### Findings

The following analysis will be conducted at three intersecting levels. First, we will employ the voices of our sample to explore the relationships between boys in the English YA system. This analysis will then be widened to examine the commodification of children in the football academy economy and how systemic violence impacts upon under par aspiring footballers, who are retained by clubs simply to facilitate the growth of a handful of players with profit-maximizing potential. Finally, our exploration will broaden in scope again to unpack what we term the academy class system, whereby the best resourced clubs’ ability to monopolise youth talent and capital ultimately supersedes all else. It is from here that we will make the argument that the YA system represents the blueprint for footballing talent identification under late capitalism and ask the question; is there a better way?

Prior to conducting this analysis, it is worth interrogating the sample’s often-unspoken complicity in the system that we will go on to dissect. Interviewees were, to a greater or lesser extent, culturally embedded in the YA system. The views we interrogate therefore represent, to borrow Scraton’s (2024: p. 195) parlance, ‘a view from above’. By this we mean that, despite speaking as individuals, the participants’ affiliations and role in the YA system may have clouded their understanding, and some sentiments likely represent the powers who ultimately pay their salaries. Similarly, many of the sample enjoyed fruitful playing careers or could at least be described as ‘football men’, having large networks of friends in the elite football industry. As such, aspects of what Lockwood (2021) describes as ‘survivor bias’ may be in effect as those who have carved out successful livelihoods in a notoriously closed industry like football may well subscribe to the dominant discourses that have shaped the game.

Similarly, despite the candid and often extremely critical sentiments that were shared with us, each interviewee engaged with a process of fetishistic disavowal. This Žižekian term refers to the active state of ‘knowing without knowing’, whereby the subject acts as if they do not know certain realities in order to continue functioning at the level of action (Winlow and Hall, 2009; Hall and Winlow, 2015). This notion is defined in the context of the workplace by Hearns-Branaman (2014: p. 21) as ‘the ability and necessity to actively critique one’s profession as long as one keeps on working’. We therefore see the sample suspend their non-belief in aspects of the YA system yet continue to play an active role in its functioning. This ultimately coheres to Žižek’s (1989) understanding that ideology exists at the level of one’s actions rather than one’s thoughts. What follows not only demonstrates the brutality of the YA system then, but also the sample’s active participation in its processes and outcomes.

### There’s no friends in football: academy relationships, amour-propre, and cold realism

It is often said that there is no such thing as friends in football. This adage is typically reserved for the elite first team environment, where seasoned professionals negotiate relationships with their coworkers in an insincere and occupationally insecure manner (Roderick, 2006a, 2006b; Jones and Denison, 2016; Adams and Darby, 2020). In this setting, Hickey and Roderick (2022) note that team ‘banter’ can be particularly cutting, diminishing true friendships and undermining genuine warmth and peer support (Thompson et al., 2018; Newman et al., 2022). But what about the youth academy, where boys are coached and educated?

Adams and Carr (2019: p. 478) examined a sample of 14- and 15-year-old YA players, exploring how their exposure to a ‘hyper-competitive, high-performance-driven ‘work-place’ context’ leads to a set of contingent relationships marred by distrust, insecurity and fierce competition. This, they argue, is a result of a cutthroat system which must pit one boy against another to instil the requisite qualities for the professional game (see also Hague and Law, 2024). Despite Ball’s (2022) contention that pockets of genuine friendship do exist across the YA system, our sample echoed Adams and Carr’s (2019) analysis, painting a picture of intense rivalry, subtly masked by camaraderie. This, we will argue, is indicative of a youth talent development model under neoliberalism.

Player Care Consultant Henry suggested that YA players are ‘*friendly with each other but also they are rivals to each other*’, which was supported by Category One Coach Tony’s statement that ‘*there’s kind of always somebody looking to take your place* […] *that’s how football is*’. Because of this, Kevin, Head of Education at a Category One YA, reflected ‘*I do not think they ever stop comparing themselves*’, capturing the perpetual self-evaluation that is fostered by the YA system, as the children internalise a belief that, if their performance drops or they incur a serious injury, they may slip from the tightrope that leads to a professional contract (Adams and Carr, 2019). Building on Roderick’s (2006a) seminal study on the working lives of professional footballers, it is evident that such a culture is embedded at a young age for aspiring players, as the systemic violence of premature professionalization (Sweeney et al., 2021; Gibbs and Briggs, 2025) casts them as self-interested individuals whose success is contingent on their teammates’ failure. Category One Coach Cameron opined:

[Competitiveness] *manifests in different forms, so we normally find at the pre-academy level it’s, “Oh so and so’s not passing me the ball,” to then first team level it’ll be, “You’re playing in my position. You’re taking food out of my mouth and you are taking money out of my pocket.”* […] *I think it’s always prevalent through the age groups*.’

However, it would be wrong to suggest that this sentiment emanates from the boys themselves. Instead, it is their willing submission to the YA system and its governing values that breeds this mindset. This was supported by Category One YA Manager, Harry:

‘*There’s certainly a camaraderie between* [YA players] *because you understand each other’s struggles and the difficulties that academy football brings when you are trying to get that pro contract. But you are well aware that you are competing against them and there’s only a set amount of contracts that can be offered ultimately*.’

Despite the camaraderie and a degree of shared struggle (Platts and Smith, 2018; Hague and Law, 2024), Harry characterises the boys’ relationships to one another as one of *amour-propre*. This Rousseauian concept describes an elevation of the self through the downfall of others (Hall et al., 2008). Put more simply, the realization of the players’ ego ideal image of sporting success – perhaps scoring a world-class goal whilst being cheered on by roaring crowds, all the while earning profane sums of money—hinges on the dereliction of the dreams of the child playing alongside them.

Winlow and Hall (2009) contend that neoliberal consumer capitalism has overseen the receding of genuine friendships, replaced with contingent instrumental ties (see also Hall and Winlow, 2005; Winlow and Hall, 2006; Telford, 2022). This appears to correspond to what our interviewees describe, as well as the wider culture of professional football (Roderick, 2006a) and other elite sporting environments (Fry and Bloyce, 2015). The tempestuousness of YA football also means that, if children do build genuine bonds with one another, these can be severed by release or even transfer.^1^ Such conditions hinder genuine, long-lasting friendships. Josh, a current professional footballer and YA graduate, recalled that ‘[y]*ou see so many players come and go, you have got friendships and then a year later you never see them again*’. More broadly, the prematurely professionalized mindset instilled in players disincentivises the playful folly of youth and rewards boys who can manage cordial, workplace-style relationships with their peers. Speaking to this, Adams and Carr (2019) note that YAs, and the football industry more generally, create an oxymoronic situation whereby the players’ main threats are also their teammates, with whom they must collaborate to ensure their future career success. Alfie, a Category Three YA coach, suggested that ‘*you absolutely are here for yourselves but if you can help your teammates get better, my god is it going to help you*’. Former professional footballer and Category One Coach Cameron highlighted how this instrumentality is baked into the industry:

‘*Listen, I played professional football. I did not get on with everyone in my squad. I did not like certain people I played with but I had to get on with it because they were a work colleague. You get that with kids as well. They’re not going to like everyone in their age group but it’s about managing yourself*.’

This imperative to ‘*manage yourself*’ speaks to an ethos of special liberty amongst YA players. Originally coined by Hall (2012a), special liberty is a state of moral and ethical exception which ‘grants the beholder the subjective permission to act as they wish, without constraint or any sense of ethical or moral obligation to the other’ (Kotzé, 2024: p. 318). Though this term has so far been reserved for serious criminality and non-sporting social harm (see Hall and Winlow, 2015; Lynes et al., 2018; Tudor, 2020; Briggs et al., 2021; Armstrong, 2025), when applied to the individualistic and hypercompetitive subjectivities described here, its pertinence becomes apparent. Despite football being a team sport, our sample painted a picture of self-interest, spanning from the pre-academy level (under-eights) through to the professional game.

Kotzé (2024) contends that special liberty, as a form of positive motivation to harm, is underpinned by two forms of self-interest: expressive and instrumental. He defines expressive self-interest as ‘a hyper-idealized expression or embodiment of neoliberal-postmodernist tenets—greed, ruthless ambition, immediate gratification, competitive individualism, liberal conceptions of freedom and a concern with status, display and the cultivation of envy in others’ (Kotzé, 2024: p. 319). This is evident in Cameron’s reduction of rival teammates as ‘*taking food out of my mouth and* […] *money out of my pocket*’, as those who can assimilate to the prevailing culture can elevate themselves above their peers and achieve the bountiful rewards on offer. Moreover, Category Three YA Head of Goalkeeping, Jordan, suggested that ‘*those players who have a bit of an edge and perhaps do not get on so well have a little bit more of an elite mindset and have a bit more competition between each other*’, arguing that such ruthless individualism is part of the ‘*elite mindset*’ sought by clubs.

Kotzé also describes *instrumental* self-interest. This is understood as ‘the pragmatic circumvention of rules, laws and conventional practices and customs. In the service of this form of self-interest, special liberty is employed to *protect the self at the expense of others*’ (Kotzé, 2024: p. 319, italics added). This helps to explain the sculpting of self-interest in YA football, as the adversarial nature of academies’ continual sieving of talent necessitates a season-long dog fight for the opportunity to progress. Key to comprehending this is an understanding that YAs are faux workplaces rather than spaces of leisure or play. Indeed, as noted by Kotzé and Antonopoulos (2023), it is often the nature of the industry or individual workplace that inspires a commitment to special liberty. Lloyd (2018: p. 107) states that ‘the unconscious motivation to act within precarious labour markets inevitably moves towards self-preservation’, diminishing ties between workers and fostering a sense of insecure competition (see Ames, 2005). We can see this clearly in the YA system as the downward pressure negates any duty to the other and, as Hall (2000, 2012a) notes, the pseudo-pacified neoliberal imperative to expel altruism plays out.

Crucially however, in line with Kotzé’s (2024) understanding that expressive and instrumental self-interest are sometimes interrelated, we can see how the boys, as transcendental materialist subjects, are not only moved by the system to adopt this mentality, but also actively solicit it as they pursue their sporting dreams in a neoliberal economy. Their mindset of special liberty therefore emanates from the YA system’s negative motivation to harm, as the systemic violence that animates the football economy creates conditions within which the boys must adopt a self-preserving instrumental self-interest to chase their sporting dreams, as well as an expressive self-interest in their overt competition with one another.

Nowhere is this tension more evident than the children’s reactions to the regular influx of trialists, who are offered up by the scouting networks that crisscross the country. As discussed by Tony:

‘*You get trialists in all the time. I do not think there’s many weeks of the season where you do not have a trialist in whatever position in each age group* […] *you have to give these kids a chance, and then they have got 8 weeks. So obviously once that trialist is done, if you do not sign him you get another one in, and if you do sign him you’ll probably still get another one in and it kind of goes like that.*’

This merry-go-round of potential replacements further entrenches the precarity of the YA faux workplace, with the children being constantly reminded of the endless conveyor belt of hungry footballers desperate to rip the shirts off their backs. Indeed, Adams and Carr (2019: p. 471) characterize the YA as ‘a setting marked by competition for places’, which was echoed by current professional footballer Josh, who stated *‘it’s almost like becoming a man, being in that environment where it can be quite ruthless at times’*. This is exacerbated by the steady drip of released players, as children who have failed to progress in Category One academies land unceremoniously on the pitches of lower Category One, Two and Three Clubs, potentially dislodging those existing players who are clinging on to their places by their fingertips. Contracted players then immediately clammer to ask, ‘*What’s his position? Is this trialist going to threaten my position?*’ (Louise, Safeguarding and Welfare Coordinator at a Category One Academy). Unsurprisingly, the influx of trialists sours relationships between the boys further, as Harry noted that:

‘*The players who are already part of it, they might be like, “Woah, what’s going on here? You cannot be coming in and trying to take my place.” They might get a bit aggressive in training, they might not speak to them. They might try and intimidate them a little bit*.’

This account of instrumental self-interest was supported by Category One Player Care Officer Jack, who recalled an under-eighteens team where ‘*one trialist came in and the other striker who was in our team was purposely out to try and kick him when he was in training*’. Jack’s example speaks to the positive motivation to harm present in some academy players, who set out to disadvantage their competition even through physical intimidation. Tony described his role in managing these tensions as one of ‘*knowing your players*’ and suggested that the insecurity of incoming trialists is necessary because ‘*they get very complacent*’ and need to ‘*have that look over their shoulder*’. This reflects a culture of cold realism (Winlow, 2012; Winlow, 2014; Ellis et al., 2017), a view that the world is a cold, hostile place and that everybody is out to tear you down unless you toughen up and defend yourself. Pulling no punches, Tony pressed on:

‘*You are going to have some horrible days, some horrible times, you are going to get injured then a player’s going to come in and take your place and you play bad for two games another player will take your place, that’s the way life is, and you have got to just be able to cope with that*.’

Similar sentiments were expressed by Jarad (Category Three club’s Player Care Lead) as he contended that fending off trialists:

‘… *Should be an opportunity for you to challenge yourself, not to down tools. I do not think it’s something you can get away from. Some players will thrive on it, some players will not. Those who are struggling will maybe struggle quite a lot more when somebody comes in*’.

Considering that this sentiment emanates from a player care professional, Jarad’s statement is striking, speaking not only to the downward pressure necessitating instrumental special liberty but also to a wider culture of cold realism in what could be termed a school of hard knocks.

### ‘Resilience’ and the training of neoliberal subjectivities

The notion of ‘resilience’ surfaced time and time again during data collection. Jarad stated, ‘…*you are going to have to have a certain resilience with competition for places* […] *you cannot just walk into a team and not be challenged otherwise it would not be a competitive sport’.* Meanwhile, Josh spoke at length about how he was hardened up by the system:

‘*The head of* [his first YA] *used this analogy of you are all sheep in a field and you go through a gate into the next field and there’s less sheep and then so on and so forth. And then by the end there’s like one or two sheep in this field. That’s a lot for an eight-year-old.* […] *That’s the thing with being in the academy system from such a young age, it builds a lot of resilience because you are exposed to that ruthless attitude and way of life from an early age, you know?* […] *it’s sort of like sink or swim.*’

‌Joseph (2013: p. 38) argues that the notion of resilience holds ‘certain ontological commitments that make it ideally suited to neoliberal forms of governance’ as it responsibilises the subject, placing the burden of coping on the individual and obfuscating the need for systemic change. Slater (2022: p. 2)similarly argues that this idea ‘fetishizes individual acts of persistence that do not pose a significant threat to the social order’, as we reward those who emerge unscathed from the buffeting storm of pseudo-pacified systemic violence. Much of this research emerged following the financial crisis and subsequent years of fiscal austerity (Mckeown and Glenn, 2018), situating terms like grit, determination, and resilience as prerequisite character traits for the neoliberal subject. Applying this, Harry reimagined the process of release, which is widely described as deleterious and distressing, stating:

*‘I actually think that on reflection if they looked at it, they would probably say that they did learn quite a lot and some of the stuff did help them. Even if it’s a negative, you know? Tough moments, having to get that news that you are not going to be retained, although it might really, really hurt at the time, that could actually help them further down the line, you know? Build up a little bit of resilience and grit and stuff.*’

This not only speaks to Gorman and Blackwood’s (2025: p. 9) recent findings that many boys are ‘surviving but not thriving’ post-release, but also the cold realist discourse noted earlier, which situates these ‘*tough moments*’ as part of a realisation that the world is an ultimately uncaring place. Of course, certain character-building experiences are necessary for healthy development, but can the wholesale demolition of boys’ footballing dreams really be justified this way? Digging deeper, Harry’s suggestion that the YA experience may ‘*help them further down the line*’ is worth examining. If, as we have argued, academies reflect the wider political economic system, might the ability to withstand the systemic violence that stimulates the sporting world serve some assimilated former players well in the broader world of work? Ethan, a former YA player who was released aged sixteen following 10 years in a Category Three academy, implicitly supported this argument as he discussed his post-university career trajectory:

‘*Since leaving* [the YA system]*, I’ve always tried to put myself in really competitive environments and I think that’s probably due to that*. […] *I look at what I chose to do for work in recruitment, which is just incredibly competitive. It is very like win, win, win, win, win. I think that probably is a carryover from being at* [YA club] *and that being the environment that I’m comfortable in.* […] *I do not think it’s a bad thing to want to win things. I do not think it’s a bad thing to be competitive.’*

Ethan cites his success in the hyper-competitive recruitment industry as a result of being ‘*comfortable*’ in such an environment following a decade of academy football. It could therefore be suggested that the culture of amour-propre, expressive and instrumental self-interest, and resilience cultivated by a prevailing sentiment of cold realism has accentuated these traits. As such, we speculatively contend that the YA is something of a training ground for neoliberal subjectivities (see Gallacher, 2019), assimilating the children in their ranks to the competitive individualist enactment of systemic violence that ensures perpetual capital growth and shareholder profit. Putting this into sharper focus, James (Head of Coaching at a Category Three YA) cited ‘*being competitive and doing what it takes to win*’ as a key transferrable skill for academy leavers. Although further research is required to substantiate any link here, those boys who can assimilate to the YA system – even if they are unable to realize their sporting aspirations – may be learning more than how to play football out on the pitches of English clubs’ academies.

Ultimately, to prosper in this hyper-competitive system ‘*you have to have a certain type of mindset*’ (Cameron). This prerequisite ‘*mindset*’ can be understood as a subjectivity cultivated by the systemic violence of neoliberalism. Cameron went on to reflect that he would be reticent to encourage his own children into the academy world ‘*because it can be quite relentless and quite ruthless at times’*. Here, we can see that Cameron’s ordinary suspension of his non-belief in the YA sector is lifted in relation to his own children, as his paternal instinct recognizes the brutal nature of the system and its role in embedding a culture of amour propre, competitive individualism and pseudo-pacified violence.

### The supporting cast model

Following our exploration of the culture of YA football, it is now worth questioning how the system views the children representing their respective clubs. Existing scholarship has identified the commodification of youth by the YA industry (Green, 2009), with boys cast as assets for clubs driven by sporting success and financial profit. Elsewhere, we have noted the practices of clubs hoarding youth talent and ‘trawling’ for prospective assets at an increasingly tender age (Gibbs and Briggs, 2025). We argued that YAs perceive the children in their care as *footballing products*, rather than potentially vulnerable children. But perhaps the best representation of this baked-in apathy towards the boys’ welfare is what we will term ‘the supporting cast’ model. By this we mean the retention of some unexceptional players in order to make up the numbers and allow the handful of valuable assets in each age group to flourish. According to Dorsett (2024), 91% of academy boys will never play a professional football match and yet thousands of children, termed ‘facilitator players’ by Gorman (2025), toil through grueling schedules, personal sacrifice, and high-pressure fixtures, laboring under the false impression that they will 1 day represent their team on the big stage. But why, given these odds, do academies retain boys who do not have a chance of making it in the professional game?

The answer to this is as simple as it is brutal: a football match requires at least twenty-two players and, for the best and brightest to succeed, they need teammates and opposition to facilitate their growth. Members of the supporting cast therefore service the developmental needs of those with genuine potential, good enough to trundle through the ranks of the YA yet lacking the talent to ‘make it’ as a professional. John (Senior Leader at a national governing body) summed this up, stating ‘*there are fifteen players in a squad to actually ensure that maybe one or two players play regularly* […] *They’ve got one or two players who they think have got a chance and* [the others] *only really exist for them’*. These boys were characterised by Chris (Category Three YA’s Head of Education) as ‘*placeholders*’, as he explained:

‘*It’s almost like players become placeholders. You need them to be a six or seven out of ten every week in order for your nines and tens to come out. Although them sixes and sevens might never get past being a six or seven, you know that they are consistently going to be in there to help your other stars of the show to shine, which is difficult*. […] *the kids probably do not understand that*.’

Despite being unaware of their function as ‘*placeholders*’, these players are effectively viewed as collateral damage, sacrificed at the footballing alter in service of a handful of golden boys. Tariq, a Category One YA Coach, noted that ‘*a lot of players in the academy system probably will not play the game to the level where they can earn enough money to feed them and be a career*’, and therefore the system actively undertakes what Liam (Category Two YA Player Care Manager) characterized as a ‘*collective individual approach*’. He continued, ‘*You’re telling everyone about the team thing but consciously, subconsciously, you are doing it for that one lad who you think, as a coach, “He’s the one”*’. Evidencing this, Tariq recalled:

*‘I’ve sat in rooms* […] *and* [other coaches] *say, “We cannot let him go because there’s no other center half,” “We cannot let him go because he’s left-footed,” “Yeah, but does he have potential?” “Not sure,” they usually say not sure rather than no because they want to keep him to facilitate* [others] *until we find a good left-footer then we are like, “No, sorry, we have got someone better, you can go.”* […] *people in the system are aware but I guess we just turn a blind eye to these kids.’*

Therefore, as Chris stated, ‘*Even though only maybe one might make it out of them 11, you need everyone else in order for that one to make it through*’. This practice bleeds into the short-term retention of trialists as stopgaps, as Liam bemoaned:

‘*You’ve got a lad in on trial and you hear the coaches talking about, “No he’s not really for me. We still really need something that’s better in that position”* […] *but then the next conversation is, “We’re running low on numbers* […] *extend his trial with us because we need him for the next few games.”*’

Liam characterized such actions as ‘*lead*[ing] *them down the garden path*’, as the systemic violence of retaining boys under the false hope of academy success is ultimately crueller than rejecting them outright. This again speaks to the negative motivation to harm that we have argued underpins much of the YA industry. By operating a neoliberal model of talent identification and development, it becomes cogent to cultivate the few to the detriment of the many, as the cold logic of capital growth and asset management cares only for those who represent future financial returns and will shear off deficient commodities only when their use value is spent. This approach is reminiscent of a space launch, as parts of the rocket are gradually jettisoned after liftoff to ultimately leave a lean, efficient machine soaring skywards. The placeholders, to continue this analogy, are ultimately discarded and land acrimoniously in the sea or scrubland below. So how many members of the supporting cast does it take to create a superstar? John used the example of England and Real Madrid ace Jude Bellingham to speculate on this:

*‘I wonder what part other players played in his development and where they are now.* […] *My guess would be a lot of them are not playing professional football.* […] *They’ve helped create Jude Bellingham, potentially at the cost of their own careers.’*

Chris similarly speculated that it takes at least thirty-five players to facilitate the growth of one academy asset, with a valuable midfielder like Bellingham relying on ‘*pretty much everyone around him*’ to ensure the maximization of his potential. However, the cost–benefit analysis implicitly undertaken by YA leadership justifies such seemingly wasteful numbers, as the supporting cast are simply the eggs that must be cracked to produce the elite sporting omelettes that we enjoy watching each week on Match of the Day.

### The haves and the have nots: the academy class system

The final and, in some sense overarching, manifestation of the YA system’s conformity to the tenets of neoliberalism is what we will term the academy class system, whose inequalities are foundational to the culture and provision of elite youth football. Following Dubal (2010), we view the elite football industry as more than just a reflection of the broader late capitalist political economy, but a magnified and loosely regulated microcosm of free market neoliberalism and corporate monopolization. It is this, we have argued elsewhere, which underpins the socially harmful practices inherent in elite boys’ football (Gibbs and Briggs, 2025).

Throughout our sample, an excruciating awareness of the inequalities of YA football, and the sport more generally, shone through. Harry stated, ‘*I think everything is getting more and more weighted to those bigger clubs and they are trying to make it that way as well, aren’t they? They’re trying to make sure that they keep all that money*.’ Similarly, Cameron expressed, ‘*I think you only have to see the way the football pyramid in this country is, it’s all geared towards the top end*’, leading to ‘*very big disparities between clubs*’ (Alan, scout for Category One YA), both within and between Categories. Indeed, our interviewees termed the best resourced clubs ‘*Category One Plus*’ (Tariq, Harry) institutions, characterised as having ‘*the best players, best facilities, best coaches, the most money*’ (Kevin). Of course, this reflects the wider domestic men’s football industry and the disproportionate influence of the so-called Big Six^2^ (‌Bishop et al., 2021), whose recent domination of English football has been dramatic. Unsurprisingly then, Cameron stated ‘*there’s a lot of inequality in terms of the Big Six will always be the Big Six and their academies will always be the Big Six academies*’. With limited exceptions, the YA system thus mirrors wider inequalities in the domestic game. Accordingly, a divide was noted between the Premier League YAs and the English Football League (EFL) academies, as Cameron conceded that:

*‘The Premier League literally run all the academies in the country. The Premier League are the academy system. I do not know if the FA can have more of a say on what happens at academies, but the FA if I’m honest they are a waste of time. They’re toothless.’*

Unable to access the riches on offer to EPL clubs, EFL clubs’ academies become relatively costly and maintaining a Category One or Two YA is extremely challenging. Going further, Cameron argued that such is the power of the biggest EPL clubs, the Big Six can ‘*hold the academy system to ransom*’ and act with relative impunity:

*‘Even the Premier League to a certain extent are toothless against the big clubs* […] *I know that* [the Big Six] *have had sanctions put against them and they have not been enforced because they said, “We will not contribute to your games programme in England. We’ve got the resources to go and play abroad so we’ll just go and play a European team every week because we can afford it. Your Games Programme will be poor because we’ll just pull out.”’*

Reminiscent of the ill-fated European Super League in 2021^3^ and indeed the Premier League breakaway in 1992,^4^ the monied clubs – and their academies – wield a great deal of power over the authorities and, in the case of the Games Programme, have the resources to whisk their boys away to play elsewhere, leaving less resourced YAs devoid of fixtures. This is similar to the sway that the super-rich have over governmental and regulatory bodies (see Østbø Kuldova et al., 2024), as the footballing ecosystem moves to the tune of these mega clubs and their will shapes the very infrastructure of the academy system. Echoing the entrenched inequalities carved out by 50 years of neoliberal capitalism then, any notion of meritocracy or possibility of a plucky less resourced academy producing a team of stars has become virtually impossible, as Cameron suggested:

‘*The best academies will always have the best players because they can find one of our under-14 s and buy him for three million* [pounds] *tomorrow if they wanted to. We could not do anything about it, do you know what I mean?* […] *if* [a lower budget academy] *got a couple of good players, they’ll just get pilfered at a young age for peanuts* […] *Where’s their chance of getting a player into their first team?*’

Echoing any number of multi-national corporations, Category One Plus YAs can simply speculate on any fledgling players with promise, spending what is for them ‘*peanuts*’ to acquire a potential superstar. This income, though measly compared with the potential return had that boy made it to the first team, is desperately needed by those at the base of the footballing pyramid. Jamie (Head of Category Two YA) spoke to this:

‘*I think now with the academy system, if a big club wants to take a player, they might take him at 11/12 when the compensation figures are less. So that sort of does hold your production line a little bit because those boys at 11 and 12, clearly talented players, you do not then get them through because they have gone already.*’

Hence, we can see the pseudo-pacified violence of the market at play, whereby Goliath beats David every time in a self-perpetuating cycle of growth and disempowerment. This negative motivation to harm is akin to a tech giant eating up various start-ups in Silicon Valley, enveloping potentially lucrative intellectual property and expertise in service of their conglomerate. In-keeping with this article’s central argument then, we can see how pseudo-pacified violence animates the YA industry and how its cultures and practices are underpinned by this free market logic.

But how does this inequality affect the boys in the system? As noted by Calvin (2017), facilities and expertise available to youth players vary dramatically depending on Category and level of resource. Whilst EPPP sets out minimum requirements for each Category around staffing and standards, some YAs may need to stretch to achieve this, whilst others can afford to go comfortably beyond the basic threshold. Recalling a recent visit to a fourth division Category Two academy, Henry stated:

‘…*They were operating on the barest of bare bones. The physiotherapist was covering three teams at one time* […] *he was a recent graduate who they’d taken from the NHS, he must have been 25, he actually fell asleep while we were at lunch because he was so tired*.’

This is the academy class system in full effect as the physiotherapy on offer to boys in this YA could be compromised compared to the large teams of medical staff described to us by staff in Category One Plus clubs. Dylan (Category Three YA Player Care Lead) summed up this tiered system, dourly noting:

‘*We cannot do anything exciting, we cannot do what* [Category One Plus YA] *do and take them all over the world* […] *and play football festivals. We simply cannot do that, we are just about planning a trip to Northern Ireland now for a pre-season trip with the 12 s-14 s, but parents are going to have to pay for it.*’

With his club sitting in League Two and having substantial financial difficulties, costs were passed onto parents and budgets could not even stretch to a pre-season tour in the United Kingdom. These limitations extend to footballing activity, as Jordan expressed:

‘*Being a Cat Three academy, we do not necessarily have all the facilities that we’d like to. So for our younger ones our goalkeeping sessions happen on a court so we have a roof, that then presents problems with longer distribution/crossing etc. But that’s just the constraints that we have. We’d love to go and hire a 3G* [astroturf pitch] *somewhere but we cannot afford that and that’s the way it is.*’

Boys in these lesser resourced institutions, just like those in lower socio-economic groups in wider society, thus receive fewer developmental opportunities, worse medical support and facilities, and could be understood as somewhat second-class citizens compared with those at the top. Of course, this is in some sense meritocratic, with the best talent, in theory, rising to the Category One clubs. But this begs the question: how many talented players has English football overlooked on account of the inequalities of this academy class system?

Thinking back to the notion of fetishistic disavowal (Black et al., 2024), these bruising inequalities were universally understood by the sample, and yet they each persisted in their roles. On this, Jordan coyly stated, ‘*the Premier League does what it wants, but it pays my wages—well some of them anyway—so I cannot complain!*’. This was similarly captured in Kevin’s resignation that YA football inequality is ‘*a microcosm of the game, is not it? The game’s not fair*’. Fundamentally, it is this unfairness that undergirds both the culture of hyper competitiveness as well as the supporting cast model, as the elite boys’ football industry functions to reinforce the Big Six monopolisation, retain a strict academy class system, and benefit those at the top of this atavistic food chain. In an overarching political economy that some have described as neo-feudal in design (Dean, 2025), can we really say that the elite football economy, and the YA system by extension, is anything but hyperconformist? Pseudo-pacified violence, we can conclude, fundamentally animates this industry.

### Is there a better way?

From the analysis offered here, we can confidently assert that English boys’ youth academies are conformist to the pseudo-pacified neoliberal economy, and the industry of talent identification and development is indelibly connected to the commodification of young players and the sculpting of neoliberal subjectivities. But what reforms and policy initiatives exist to protect children in the system? And how effective are they against the cruelties explored in this paper?

In 2019, the EPPP mandated the employment of at least one full-time player care professional in every Category One, Two, and Three academy (Premier League, 2022). These practitioners, some of whom were interviewed for this project, work collaboratively with organizations like League Football Education to provide life skills training, offer pastoral support throughout the academy journey, and lead release and aftercare provision. It was universally acknowledged by the sample that this resource—which includes best practice like academy alumni programmes, support for released players seeking trials elsewhere, and follow-up phone calls post-deselection—has vastly improved in the last decade. However, mindful of the systemic challenges highlighted in this article, we question whether such well-minded interventions can shield the boys from the harms of the YA industry. To this end, it is worth introducing two interrelated critical frameworks capable of interrogating the effectiveness of current provision and signposting a path to meaningful change: Stevens et al.’s (2025) idea of ‘interventionitis’ and Raymen’s (2023) ‘assumption of harmlessness’.

Stevens et al. (2025: p. 1)define interventionitis as ‘the tendency of policymakers to treat enduring, systemically generated problems with limited interventions that are insufficient or inappropriate for the intended improvement’. Arguably, the promise to address the deeply embedded systemic violence explored in this work with the mandating of player care and a handful of adjacent initiatives satisfies this definition. We therefore understand these well-meaning interventions as ‘another way of failing to attend to […] structural and historical’ issues (Stevens et al., 2025: p. 2) and read these attempts as akin to tackling a forest fire with a garden hose. Player care, and the goodwilled practitioners tasked with carrying it out, treats the symptoms of the pseudo-pacified violent YA industry rather than addressing the root cause. This notion is fleshed out by Raymen (2023: p. 57) in his concept of the assumption of harmlessness, whereby surface-level interventions satiate our need to act against the perceived harms of late capitalist industries, without threatening the system itself:

‘There are, we point out, individuals and organizations out there who are ironing-out the harms and the kinks in the system. There is no need to press for wholesale political-economic change. […] No need to confront the obscene real of capitalism too closely or deliberate too deeply over what should be the proper shared goals and ends of life. We just need to develop better tools to identify those who are vulnerable and at risk and develop strategies that can mitigate the worst excesses of their problems. Rather than being recognised as symptoms of deep social problems that are to be resolved by ambitious theoretical work that attempts to re-imagine a different ethical, political, and economic basis for society, social harms have become transformed into risks to be managed’ (Raymen, 2023: pp. 57–58).

We can therefore comprehend the recent reforms as conformist mitigation of the excesses of harm and pseudo-pacified violence, the equivalent of offering an opposition player a hand up after a particularly crunching slide tackle. This approach, described by Raymen (*ibid*.) as a ‘post-political fetishisation of piecemeal harm-reduction and harm-minimization’, poses no ideological or structural challenge to the forces that we have argued animate the cultural, procedural, and systemic issues at play. Instead, player care initiatives tinker at the edges, allowing those in power to interpassively ‘look busy’ without any attempt to dismantle the harmful apparatus driving the issues at play.

How then can we enact systemic change in an industry so charged with pseudo-pacified systemic violence? Elsewhere, we have advocated for an academy model based on the notion of ‘mattering’ (Gibbs and Briggs, 2025; Billingham and Irwin-Rogers, 2022) wherein the holistic wellbeing of children should take precedence over their prospective economic value and the focus, certainly in the Foundation phase, should be on fun and human flourishing alongside the craft of football. Interestingly, several of our sample expressed a belief that academies ought not to recruit boys at the tender age of 9 (or earlier, in the case of pre-academy programmes) and should instead ‘*let youngsters be youngsters*’ (Jamie). This sentiment was expressed by Cameron: ‘*I would not have kids anywhere near academies until they were at least 12. I just do not see the need for it, and I think they need to enjoy their football as long as they can.*’ As documented by Mac Lelland (2020), German superclub Bayern Munich recently phased out their under-nine and tens teams, citing the benefits of players enjoying a less pressurised childhood and enhanced creative freedom (Austin, 2020). Could mandating later recruitment of players work to salvage the childhoods of boys in the system, and protect them from the culture and practices of systemic violence? However, this suggestion itself could be construed as another example of ‘over-optimistic faith in the capacity of limited and unevidenced interventions to resolve systemically driven problems’ (Stevens et al., 2025: p. 10) and not the structural change necessitated by the structural issues under investigation here.

We therefore advocate for something more radical. Given the gross inequalities between clubs and the dominance of the Premier League, we argue that funding ought to be centralized by a more benign body, perhaps the FA or a newly created organization, with finance sourced from a means-tested levy on all 92 clubs to create an equitable landscape, counteracting the current academy class system. Within this, initial recruitment ought to be conducted with a focus on quality over quantity (see EFL, 2024) so as to negate the hoarding of young players by bigger academies (Gibbs and Briggs, 2025) and the volume of ‘placeholder’ players in the system. This recruitment, we argue, should happen no earlier than aged 12, and boys should be encouraged to play grassroots football until then. Similarly, to counter the issue of less resourced academies losing out on their most talented players through transfers to clubs with more clout, transfer activity—excluding extreme cases—should be outlawed and talented boys should be given the opportunity to cut their teeth for their academy’s senior team to ensure proper renumeration for their parent club. Despite Cameron’s pessimistic suggestion that ‘*the big clubs will find a way of getting hold of the kids anyway*’, this rule would allow each club to claim the appropriate value of the player and, more importantly, would delay the player’s inevitable commodification until adulthood.

Of course, these reforms, however radical, cannot undo the state of capitalist realism (Fisher, 2009) that has a stranglehold on the YA industry. They may also struggle to disassemble the culture of amour-propre and cold realism documented by our sample. We are of course also not naïve enough to dismiss the need for selectivity as a requisite for elite sport. Not all YA players can achieve ‘every boy’s dream’ (Green, 2009) of becoming professional footballers, but the supporting cast model and its inherent dishonesty cannot remain the blueprint for development and release. However, we must conclude on a rather melancholic note. If we are to move beyond the current paradigm of talent identification, ruthless individualism, and vast financial inequalities between clubs, we would require an ‘epochal and progressive shift away from neoliberalism’ (Telford, 2024: p. 401). Following Winlow (2025), we do not hold out much hope of such change and, as long as the neoliberal game continues on this course, the boys funneled through youth academies may have to rely on a system of mitigation whose diagnosis of interventionitis is terminal.

## Conclusion

This article has made a significant and original contribution to the social scientific literature on boys’ youth academy football through an exploration of the systemic violence present in the YA industry. First documenting the culture of amour propre and cold realism alongside academies’ status as breeding grounds for neoliberal subjectivities, we have argued that pseudo-pacified violence fundamentally animates the industry, instilling a positive motivation to harm in the form of expressive and instrumental self-interest. We have also highlighted the ‘supporting cast’ model as a product of a system that cannot see beyond the economic worth of a child and instead treats the boys as assets, coldly jettisoning them when their use value is spent. Underpinning this, we have argued, is an academy class system that mirrors the broader inequalities and monopolies of late capitalism, where the Big Six and the EPL dominate smaller clubs to perpetuate their own advantage.

Leaning on the work of Raymen (2023) and Stevens et al. (2025), we then argued that to meaningfully address the brutalities presented, we must move beyond the current interventionitis approach and instead seek wholesale change. We hope that this work has, to quote Raymen (2023: p. 57), spotlighted the ‘need to confront the obscene real of capitalism’ baked into the youth academy industry and emphasized the necessity to recognize the previously overlooked systemic violence that paves the pathway to professional men’s football.

## Data Availability

The datasets presented in this article are not readily available because research participants did not give consent to share data publicly. Requests to access the datasets should be directed to NG at n.gibbs@northumbria.ac.uk.

## Footnotes

^1^In some circumstances, players can move between academies, with compensation paid to the former club (see Premier League, 2024).

^2^Generally, the ‘Big Six’, though subject to change season by season are made up of Manchester City, Liverpool FC, Chelsea FC, Manchester United, Tottenham Hotspur, and Arsenal.

^3^This was an attempt to form a new elite European league to rival the Champions League, featuring teams like Real Madrid, Juventus FC, Manchester United and Barcelona FC, which was met with vehement opposition when it was announced in 2021 and subsequently abandoned.

^4^For an accessible account of this, please see Clegg and Robinson (2018).

## References

[ref1] Adams A. Carr S. (2019). Football friends: adolescent boys’ friendships inside an English professional football (soccer) academy. Soccer Soc. 20, 471–493. doi: 10.1080/14660970.2017.1331164

[ref2] Adams R. Darby P. (2020). Precarious pursuits, broken ‘dreams’ and immobility among northern Irish soccer migrants. Sport Soc. 23, 920–937. doi: 10.1080/17430437.2019.1593376

[ref9001] Ames M. (2005). Going Postal. New York: Soft Skull.

[ref3] Armstrong E. (2025). British Army veterans’ experiences of the transition into civilian life. Abingdon: Taylor & Francis.

[ref4] Atkinson C. (2022). ‘Football fans are not thugs’: communication and the future of fan engagement in the policing of Scottish football. Polic. Soc. 32, 472–488. doi: 10.1080/10439463.2021.1916491

[ref5] Austin S. (2020). *Bayern Munich to scrap U9 and U10 teams*. [online] training ground guru. Available online at: http://archive.trainingground.guru/articles/bayern-munich-to-scrap-u9-and-u10-teams (Accessed April 29, 2025).

[ref6] Awan I. Zempi I. (2023). Hate crime in football. Bristol: Bristol University Press.

[ref7] Ball D. (2022). From winning teams to broken dreams: The story of six friends and their journey to make to the premier league: Self-published. Available at: https://www.amazon.co.uk/Winning-Teams-Broken-Dreams-friends/dp/B09XZ162HG#:~:text=A%20book%20every%20parent%20should,to%20become%20Premier%20League%20footballers

[ref8] Billingham L. Irwin-Rogers K. (2022). Against youth violence: A social harm perspective. Bristol: Policy Press.

[ref9] ‌Bishop R. Smith A. C. T. Read D. (2021). An introduction to the challenges of distributive equity in the English premier league. Sport Bus. Manag. 12, 284–304. doi: 10.1108/sbm-04-2021-0053, PMID: 35579975

[ref10] Black J. Sinclair G. Kearns C. (2024). The fetishization of sport: exploring the effects of fetishistic disavowal in sportswashing. J. Sport Soc. Issues 48, 145–164. doi: 10.1177/01937235241269906, PMID: 40735054

[ref11] Blakelock D. J. Chen M. A. Prescott T. (2016). Psychological distress in elite adolescent soccer players following deselection. J. Clinic. Sport Psychol. 10, 59–77. doi: 10.1123/jcsp.2015-0010

[ref12] Blakelock D. Chen M. Prescott T. (2019). Coping and psychological distress in elite adolescent soccer players following professional academy deselection. J. Sport Behav. 41, 1–26.

[ref13] Bradbury S. Conricode D. (2020). “Addressing the under-representation of BAME coaches: an examination of the EFL mandatory code of coach recruitment in professional football club youth academies” in Sport coaching with diverse populations: Theory and practice. eds. Wallis J. Lambert J. (London: Routledge), 100–112.

[ref14] Bradbury S. Conricode D. (2024). Racialisation and the inequitable experiences of racialised minority coaches in men’s professional football club youth academies in England. Int. Rev. Sociol. Sport 60, 251–277. doi: 10.1177/10126902241266613, PMID: 40735054

[ref15] Briggs D. Telford L. Lloyd A. Ellis A. Kotzé J. (2021). Lockdown: Social harm in the Covid-19 era. Cham: Palgrave Macmillan.

[ref16] Brooks-Hay O. Lombard N. (2018). ‘Home game’: domestic abuse and football. J. Gender Based Viol. 2, 93–108. doi: 10.1332/239868018X15155986580769, PMID: 40557707

[ref17] Brown G. Potrac P. (2009). ‘You’ve not made the grade, son’: de-selection and identity disruption in elite level youth football. Soccer & Society 10, 143–159. doi: 10.1080/14660970802601613

[ref18] Bullough S. Jordan J. (2017). Youth academy player development in English football. Sport Bus. Manag. 7, 375–392. doi: 10.1108/sbm-10-2016-0059, PMID: 35579975

[ref19] Calvin M. (2017). No hunger in paradise: The players. The journey. The dream. London: Random House.

[ref21] Champ F. Ronkainen N. Nesti M. S. Tod D. Littlewood M. (2020). ‘Through the lens of ethnography’: perceptions, challenges, and experiences of an early career practitioner-researcher in professional football. Qual. Res. Sport, Exerc. Health 12, 513–529. doi: 10.1080/2159676x.2019.1638444, PMID: 40640861

[ref22] CIES. (2024). Most profitable club academies worldwide—CIES football observatory. Available online at: https://football-observatory.com/WeeklyPost446 (Accessed April 12, 2025).

[ref23] Clegg J. Robinson J. (2018). The Club: How the premier league became the richest, Most disruptive business in sport. London. John Murray.

[ref24] Dean J. (2025). Capital's grave: Neofeudalism and the new class struggle. London: Verso.

[ref25] DeKeseredy W. S. (2025). The critical criminology of sport: past, present, future. J. Criminol. doi: 10.1177/26338076251341580

[ref26] Dicce R. Ewers M. (2021). “Becoming linked in: leveraging professional networks for elite surveys and interviews” in *Geographical fieldwork in the 21st Centur*y. eds. McSweeney K. WinklerPins A. (London: Routledge), 160–171.

[ref27] Dixon K. (2020). “Sexual abuse and masculine cultures: reflections on the British football scandal of 2016” in The Palgrave handbook of masculinity and sport. eds. Magrath R. Cleland J. Anderson E. (London: Palgrave Macmillan), 73–93.

[ref28] Dorsett R. (2024). The route to the top. [online] Sky Sports. Available online at: https://www.skysports.com/football/story-telling/11095/13257286/chasing-the-dream-trent-alexander-arnold-and-kyle-walker-discuss-the-embarrassing-problem-in-academy-football (Accessed January 17, 2025).

[ref29] Dubal S. (2010). The neoliberalization of football: rethinking neoliberalism through the commercialization of the beautiful game. Int. Rev. Soc. Sport 45, 123–146. doi: 10.1177/1012690210362426

[ref31] EFL. (2024) Parents & carers handbook 2024–2025. [online] Available online at: https://s3.eu-west-1.amazonaws.com/gc-media-assets.gc.eflservices.co.uk/0dc34f00-6156-11ef-9b47-e7819675458d.pdf [Accessed December 12, 2024).

[ref32] Ellis A. Winlow S. Hall S. (2017). ‘Throughout my life I’ve had people walk all over me’: trauma in the lives of violent men. Sociol. Rev. 65, 699–713. doi: 10.1177/0038026117695486

[ref33] Fisher M. (2009). Capitalist realism: Is there no alternative? Alresford: Zero Books.

[ref34] Fry J. Bloyce D. (2015). ‘Friends as enemies’: A sociological analysis of the relationship among touring professional golfers. Int. Rev. Sociol. Sport 52, 336–360. doi: 10.1177/1012690215597659, PMID: 40735054

[ref35] Gallacher G. (2019). “Sporting success? A critical criminology of children’s grassroots football” in A tutto campo’: ricerche, intrecci, riflessioni su sport e criminalità. eds. Altopiedi R. Verga M. (Rome: Antigone), 11–22.

[ref36] Gallacher G. (2022). “The (De)civilizing process: an ultra-realist examination of sport” in Power played: A critical criminology of sport. eds. Silva D. Kennedy L. (Vancouver: UBC Press), 74–100.

[ref37] Gibbons T. Dixon K. Braye S. (2008). ‘The way it was’: an account of soccer violence in the 1980s. Soccer Soc. 9, 28–41. doi: 10.1080/14660970701616704

[ref38] Gibbs N. Briggs D. (2025). “Relegating football dreams: the elite boys’ football youth academy system as a harm facilitative industry” in Criminology, leisure, and sport: Interdisciplinary perspectives. eds. Berry M. Berry C. Caudwell J. (London: Routledge).

[ref39] Gorman J. (2025). A footballer dies twice. [online]. Available online at: https://johnnygorman.substack.com/p/a-footballer-dies-twice?r=q8egx&utm_medium=ios&triedRedirect=true (Accessed April 23, 2025).

[ref40] Gorman J. Blackwood L. (2025). Inside the football factory: young players’ reflections on being ‘released’. Qualit. Res. Sport Exerc. Health 17, 47–60. doi: 10.1080/2159676x.2024.2406199

[ref41] Green C. (2009). Every boy’s dream: England’s football future on the line. London: A. & C. Black.

[ref42] Groombridge N. (2016). Sports criminology: A critical criminology of sport and games. Bristol: Policy Press.

[ref43] Hague N. Law G. (2024). ‘We’re sweeping the floors’: life as an EFL championship youth academy player. Soccer Soc. 25, 272–288. doi: 10.1080/14660970.2023.2248000, PMID: 40640861

[ref44] Hall S. (2000). Paths to Anelpis: 1: dimorphic violence and the pseudo-pacification process. Parallax 6, 36–53. doi: 10.1080/13534640050083783

[ref45] Hall S. (2012a). Theorizing crime and deviance: A new perspective. London: Sage.

[ref46] Hall S. (2012b). The solicitation of the trap: on transcendence and transcendental materialism in advanced consumer-capitalism. Hum. Stud. 35, 365–381. doi: 10.1007/s10746-012-9246-9

[ref47] Hall S. Ancrum C. Winlow S. (2008). Criminal identities and consumer culture: Crime, exclusion and the new culture of narcissism. Abingdon: Routledge.

[ref48] Hall S. Winlow S. (2005). Anti-nirvana: crime, culture and instrumentalism in the age of insecurity. Crime Media Culture 1, 31–48. doi: 10.1177/1741659005050242

[ref49] Hall S. Winlow S. (2015). Revitalizing criminological theory: Towards a new UltraRealism. London: Routledge.

[ref50] Hearns-Branaman J. O. (2014). Journalistic professionalism as indirect control and fetishistic disavowal. Journalism 15, 21–36. doi: 10.1177/1464884912474202

[ref51] Hickey C. Roderick M. (2022). When jokes aren’t funny: banter and abuse in the everyday work environments of professional football. Eur. Sport Manag. Q. 24, 383–403. doi: 10.1080/16184742.2022.2124299, PMID: 40640861

[ref52] Hopkins M. Treadwell J. (2014). Football hooliganism, fan behaviour and crime. London: Palgrave MacMillan.

[ref53] Horsley M. Kotzé J. Hall S. (2015). The maintenance of orderly disorder: Law, markets and the pseudo-pacification process. J. Europ. Hist. Law 6, 18–29.

[ref54] Jones L. Denison J. (2016). Challenge and relief: A Foucauldian disciplinary analysis of retirement from professional association football in the United Kingdom. Int. Rev. Sociol. Sport 52, 924–939. doi: 10.1177/1012690215625348, PMID: 40735054

[ref55] ‌Joseph J. (2013). Resilience as embedded neoliberalism: a governmentality approach. Resilience 1, 38–52. doi: 10.1080/21693293.2013.765741

[ref56] Kotzé J. (2024). On special liberty and the motivation to harm. Br. J. Criminol. 65, 314–327. doi: 10.1093/bjc/azae053, PMID: 33061623

[ref57] Kotzé J. Antonopoulos G. A. (2023). Con air: exploring the trade in counterfeit and unapproved aircraft parts. Br. J. Criminol. 63, 1293–1308. doi: 10.1093/bjc/azac089

[ref58] Larkin P. Reeves M. J. (2018). Junior-elite football: time to re-position talent identification? Soccer Soc. 19, 1–10. doi: 10.1080/14660970.2018.1432389, PMID: 40640861

[ref59] Law G. (2019). Researching professional footballers: reflections and lessons learned. *International journal of qualitative*. Methods 18:160940691984932. doi: 10.1177/1609406919849328, PMID: 40735054

[ref60] Lloyd A. (2018). The harms of work: An ultra-realist account of the service economy. Bristol: Bristol University Press.

[ref61] Lockwood D. (2021). Fooled by the winners. Austin, Texas: Greenleaf Book Group.

[ref62] Lynes A. Kelly C. Uppal P. K. S. (2018). Benjamin’s ‘flâneur’ and serial murder: an ultra-realist literary case study of Levi Bellfield. Crime Media Culture 15, 523–543. doi: 10.1177/1741659018815934, PMID: 40735054

[ref63] Lynes A. Treadwell J. Bavin K. (2024). Crimes of the powerful and the contemporary condition: The Democratic Republic of capitalism. Bristol: Bristol University Press.

[ref64] Mac Lelland G. (2020). Bayern Munich scrap U9 and U10 academy teams – A positive move for youth sport or not? – Working with Parents in Sport. [online] Parentsinsport.co.uk. Available online at: https://www.parentsinsport.co.uk/2020/05/03/bayern-munich-scrap-u9-and-u10-academy-teams-a-positive-move-for-youth-sport-or-not/ (Accessed April 29, 2025).

[ref65] McGlinchey T. R. Saward C. Healy L. C. Sarkar M. (2022). ‘From everything to nothing in a split second’: elite youth players’ experiences of release from professional football academies. Front. Sports and active Living 4:941482. doi: 10.3389/fspor.2022.941482, PMID: 35935062 PMC9354464

[ref66] Mckeown A. Glenn J. (2018). The rise of resilience after the financial crises: a case of neoliberalism rebooted? Rev. Int. Stud. 44, 193–214. doi: 10.1017/s0260210517000493

[ref67] Millward P. Ludvigsen J. A. L. Sly J. (2022). Sport and crime: Towards a critical criminology of sport. Abingdon, Oxon: Routledge.

[ref68] ‌Moore N. Stokes P. (2012). Elite interviewing and the role of sector context: an organizational case from the football industry. Qual. Mark. Res. Int. J. 15, 438–464. doi: 10.1108/13522751211257105, PMID: 35579975

[ref69] Newman J. A. Warburton V. E. Russell K. (2022). It can be a ‘very fine line’: professional footballers’ perceptions of the conceptual divide between bullying and banter. Front. Psychol. 13:838053. doi: 10.3389/fpsyg.2022.838053, PMID: 35282241 PMC8908421

[ref70] O’Gorman J. Partington M. Potrac P. Nelson L. (2021). Translation, intensification and fabrication: professional football academy coaches’ enactment of the elite player performance plan. Sport Educ. Soc. 26, 309–325. doi: 10.1080/13573322.2020.1726313

[ref71] Østbø Kuldova T. Østbø J. Raymen T. (2024). Luxury and corruption: Challenging the anti-corruption consensus. Bristol: Policy Press.

[ref72] Platts C. Smith A. (2018). ‘We don’t need no education’? Exploring the educational experiences of young footballers. Br. J. Sociol. Educ. 39, 925–941. doi: 10.1080/01425692.2018.1434408

[ref73] Premier League (2011) *Elite player performance plan*. [online] Available online at: https://www.goalreports.com/EPLPlan.pdf (Accessed February 2, 2025).

[ref74] Premier League. (2022). *Key facts about the impact of the EPPP*. [online] Available online at: https://www.premierleague.com/news/2911916 (Accessed March 16, 2025).

[ref75] Premier League. (2024). Parent hub - rights, Rules and Responsibilities. [online] Available online at: https://www.premierleague.com/news/4018229 (Accessed December 16, 2024).

[ref76] Raymen T. (2023). The enigma of social harm: The problem of liberalism. London: Routledge.

[ref77] Roderick M. (2006a). The work of professional football: A labour of love? New York, NY: Routledge.

[ref78] Roderick M. (2006b). A very precarious profession: uncertainty in the working lives of professional footballers. Work Employ. Soc. 20, 245–265. doi: 10.1177/0950017006064113

[ref80] Scraton P. (2024). 21st Kilbrandon lecture. Scottish J. Resident. Child Care 23, 189–220. doi: 10.17868/strath.00089084

[ref81] ‌Silva D. Kennedy L. (2022a). Power played: a critical criminology of sport, Vancouver: UBC Press

[ref82] Silva D. Kennedy L. (2022b). “Introduction: toward a critical criminology of sport” in Power played: A critical criminology of sport. eds. Silva D. Kennedy L. (Vancouver: UBC Press), 3–45.

[ref83] Slater G. (2022). Terms of endurance: resilience, grit, and the cultural politics of neoliberal education. Crit. Educ. 13, 1–16. doi: 10.14288/ce.v13i1.186530

[ref84] Stevens A. Schreeche-Powell E. Billingham L. Irwin-Rogers K. (2025). Interventionitis in the criminal justice system: three English cases. Crit. Criminol. 33, 171–187. doi: 10.1007/s10612-024-09808-x

[ref85] Sweeney L. Horan D. MacNamara Á. (2021). Premature professionalisation or early engagement? Examining Practise in football player pathways. Front Sports Active Living 3:167. doi: 10.3389/fspor.2021.660167, PMID: 34164620 PMC8215134

[ref86] Tears C. Chesterton P. Wijnbergen M. (2018). The elite player performance plan: the impact of a new national youth development strategy on injury characteristics in a premier league football academy. J. Sports Sci. 36, 2181–2188. doi: 10.1080/02640414.2018.1443746, PMID: 29478360

[ref87] Telford L. (2022). English nationalism and its ghost towns. Abingdon: Taylor & Francis.

[ref9002] Telford L. (2024). Neoliberalism, Left behind Middlesbrough and Levelling up: an Intractable Policy task?. Contemp. Soc. Sci. 19, 1–23. doi: 10.1080/21582041.2024.2408252

[ref88] Telford L. Lloyd A. (2020). From ‘infant Hercules’ to ‘ghost town’: industrial collapse and social harm in Teesside. Crit. Criminol. 28, 595–611. doi: 10.1007/s10612-020-09523-3

[ref89] Thompson J. Johnstone J. Banks C. (2018). An examination of initiation rituals in a UK sporting institution and the impact on group development. Eur. Sport Manag. Q. 18, 544–562. doi: 10.1080/16184742.2018.1439984

[ref90] Tudor K. (2020). “Toxic sovereignty: understanding fraud as the expression of special liberty within late capitalism” in Crime, harm and consumerism. eds. Hall S. Kuldova T. Horsley M. (London: Routledge), 140–155.

[ref91] Webb T. Rayner M. Cleland J. O’Gorman J. (2020). Referees, match officials and abuse: Research and implications for policy. London: Routledge.

[ref92] Winlow S. (2012). “‘All that is sacred is profaned’: towards a theory of subjective violence” in New directions in criminological theory. eds. Hall S. Winlow S. (London, UK: Routledge), 199–215.

[ref93] Winlow S. (2014). Trauma, guilt and the unconscious: some theoretical notes on violent subjectivity. Sociol. Rev. 62, 32–49. doi: 10.1111/1467-954x.12190

[ref94] Winlow S. (2025). The politics of nostalgia: Class, rootlessness and decline. Leeds: Emerald Group Publishing.

[ref95] Winlow S. Hall S. (2006). Violent night: Urban leisure and contemporary culture. Oxford: Berg.

[ref96] Winlow S. Hall S. (2009). Living for the weekend: youth identities in Northeast England. Ethnography 10, 91–113. doi: 10.1177/1466138108099588

[ref9003] Wilkinson R. J. (2021). A literature review exploring the mental health issues in academy football players following career termination due to deselection or injury and how counselling could support future players. Couns. Psychother. Res. 21. doi: 10.1002/capr.12417

[ref98] Young J. (2011). The criminological imagination. Cambridge: Polity Press.

[ref99] Žižek S. (1989). The sublime object of ideology. London: Verso.

[ref100] Žižek S. (2008). Violence: Six sideways reflections. London: Picador.

